# Comparative genomics of a poinsettia-associated phytoplasma and functional validation of its SAP11-homologous effectors that induce plant branching

**DOI:** 10.1099/mgen.0.001675

**Published:** 2026-03-30

**Authors:** Shen-Chian Pei, Nian-Pu Li, Ting-Ting Li, Ya-Ching Yang, Ting-Hsuan Hung, Chih-Horng Kuo

**Affiliations:** 1 Institute of Plant and Microbial Biology, Academia Sinica, Taipei 115201, Taiwan, ROC; 2 Department of Plant Pathology and Microbiology, National Taiwan University, Taipei 106319, Taiwan, ROC; 3 Taoyuan District Agricultural Research and Extension Station - Shulin Substation, Ministry of Agriculture, New Taipei 238014, Taiwan, ROC

**Keywords:** effector, genomics, phytoplasma, plant pathogen, poinsettia

## Abstract

Phytoplasmas are insect-transmitted plant pathogens that manipulate host development through secreted effector proteins. While they are notorious for causing agricultural losses, in the ornamental plant poinsettia (*Euphorbia pulcherrima*), phytoplasma infection is uniquely harnessed to induce the commercially desirable free-branching trait. However, the effectors responsible for this phenotype have remained unknown. To address this question, we sequenced and analysed the 705,138 bp genome of ‘*Candidatus* Phytoplasma pruni’ PR2021, a strain associated with the high-branching cultivar Princettia Pink. Comparative genomics confirmed its species assignment and revealed an unusual effector repertoire. PR2021 lacks most previously described effectors but encodes two distinct SAP11 homologues, a family of effectors known to induce plant branching. Genomic context analysis showed that one homologue is located within a potential mobile unit (PMU) and is nearly identical to the SAP11 of the distantly related ‘*Ca*. P. asteris’, while the other is located outside PMU regions and is divergent in protein sequence (39.0% identity) and predicted structure. Functional assays using *Agrobacterium*-mediated transient expression in *Nicotiana benthamiana* demonstrated that each homologue independently induced significant branching, whereas co-expression did not enhance the phenotype, indicating overlapping functions. These findings establish a direct connection between poinsettia branching and SAP11-homologous effectors, providing the first experimental evidence linking phytoplasma effector activity to this horticulturally important trait. This work expands understanding of phytoplasma effector diversity and mobility, while offering a functional framework for developing pathogen-free strategies to modulate ornamental plant architecture.

**Impact Statement**

Phytoplasmas are uncultivated bacterial pathogens that reprogram host development through secreted effectors. While they are notorious for causing agricultural losses, phytoplasma infection is uniquely harnessed to induce the desirable free-branching trait in poinsettia, although the molecular basis has remained unresolved. Through analysis of the complete genome of ‘*Candidatus* Phytoplasma pruni’ PR2021, a strain associated with a high-branching cultivar, we identified two SAP11-homologous effectors with contrasting genomic and evolutionary contexts. One appears vertically inherited and divergent from previously characterized homologues, whereas the other is embedded in a potential mobile unit and likely acquired through horizontal transfer. Importantly, both homologues induce branching despite substantial sequence divergence. Taken together, this work advances understanding of phytoplasma genome evolution and effector diversity, while providing experimental evidence that links effector function to host developmental manipulation. Beyond its horticultural relevance, it illustrates how horizontal gene transfer and lineage-specific retention shape phytoplasma effector complements, offering a foundation for future efforts to dissect and re-engineer effector–host interactions.

## Data Summary

All genome assemblies analysed in this study were obtained from the National Center for Biotechnology Information (NCBI) genome database. The accession numbers are provided in Table S2, available in the online Supplementary Material 1.

## Introduction

Phytoplasmas are insect-transmitted plant pathogens that infect >1,000 plant species [1,4]. These uncultivated bacteria are classified into more than 40 species in the genus ‘*Candidatus* Phytoplasma’ (‘*Ca*. P.’) [5,6]. Similar to other related lineages in the phylum *Mycoplasmatota*, phytoplasmas are characterized by a lack of cell wall, small cell size and reductive genomes with low GC content [7]. However, phytoplasmas are unique among *Mycoplasmatota* genera in their ability to manipulate the development programmes of infected plants, causing symptoms such as stunting (shortening of internodes, reducing the size of leaves and flowers), witches’ broom (proliferation of stems and leaves), virescence (greening of flowers) and phyllody (abnormal development of floral parts into leaf-like tissues).

These abnormalities of host development are modulated through small secreted proteins produced by phytoplasmas known as effectors. Notable examples include (1) SAP05, which induces witches’ broom and prolongs the host lifespan [8]; (2) SAP06, which stunts vegetative growth and increases seed dormancy [9]; (3) SAP11/SWP11, which alters the morphogenesis of leaves and roots, phosphate starvation response and defence response [10,17]; (4) SAP54/PHYL1, which induces leaf-like flower development and promotes insect colonization on the plants [17,23]; and (5) TENGU, which causes witches’ broom and stunting [24,25], as well as down-regulation of jasmonic acid and auxin pathways to result in plant sterility [26]. It is worth noting that all of these effectors were initially characterized in ‘*Ca*. P. asteris’ and some were later studied in other ‘*Ca*. P.’ species. The presence/absence patterns regarding the homologous genes of these effectors are highly variable among different ‘*Ca*. P.’ species and strains [27].

The ability to manipulate plant development, coupled with their wide host range and geographic distribution, made phytoplasmas serious threats to agriculture [1,3]. Intriguingly, in one notable exception, their unique ability to modulate plant development is harnessed for the production of an ornamental plant. In this case, the bushy growth and dwarfism induced by phytoplasma infection are favourable traits of poinsettia (*Euphorbia pulcherrima*), resulting in many axillary shoots and ‘flowers’ (modified leaves with bright red/pink colours called ‘bracts’) [28]. This ‘free-branching’ phenotype is important for large-scale production of vegetative cuttings and also increases the commercial value. Therefore, the poinsettia branch-inducing (PoiBI) phytoplasmas associated with such a trait have been integrated into most commercial poinsettia cultivars. Reflecting this importance, the annual market value of potted poinsettias in the USA is approximately $157 million USD [29]

Recent studies of this poinsettia-phytoplasma system demonstrated that phytoplasma titre in stock plants was positively correlated with the branching phenotype of propagated cuttings, and higher phytoplasma loads were consistently detected in source leaves from the lower parts of the plant [30]. In addition, the expression of the poinsettia phenylalanine ammonia-lyase gene (*EpPAL*) varied among tissues and was negatively correlated with phytoplasma titre, suggesting that *EpPAL*-linked defence pathways may restrict pathogen growth [31]. However, the exact phytoplasma effectors associated with the poinsettia branching phenotype remained unknown. Identifying these effectors is not only critical for explaining the poinsettia branching phenotype but also offers broader opportunities to link effector function with mechanisms of plant development. In the long term, such insights may enable the replacement of phytoplasmas, which can be highly variable, with breeding strategies or synthetic effector mimics to achieve desirable traits.

To address the question of which phytoplasma effectors underlie the branch-inducing phenotype observed in poinsettia, we conducted metagenomic shotgun sequencing of the poinsettia cultivar Princettia Pink. The complete genome sequence of phytoplasma strain PR2021 associated with this cultivar was determined using a hybrid assembly method that combined Illumina and Oxford Nanopore Technologies (ONT) sequencing results. To provide early data access for the research community, the genome sequence of PR2021 has been made publicly available and published as a genome announcement [32]. In this work, we present the branching phenotype comparison among poinsettia cultivars that led to the selection of Princettia Pink as the target of our sequencing effort, detailed analysis of the PR2021 genome and comparisons with other phytoplasmas and experimental validation of two distinct SAP11 homologues from PR2021 that can induce branching phenotype in the model plant *Nicotiana benthamiana*.

## Methods

### Quantification of branching performance among poinsettia cultivars

**Fig. 1.**
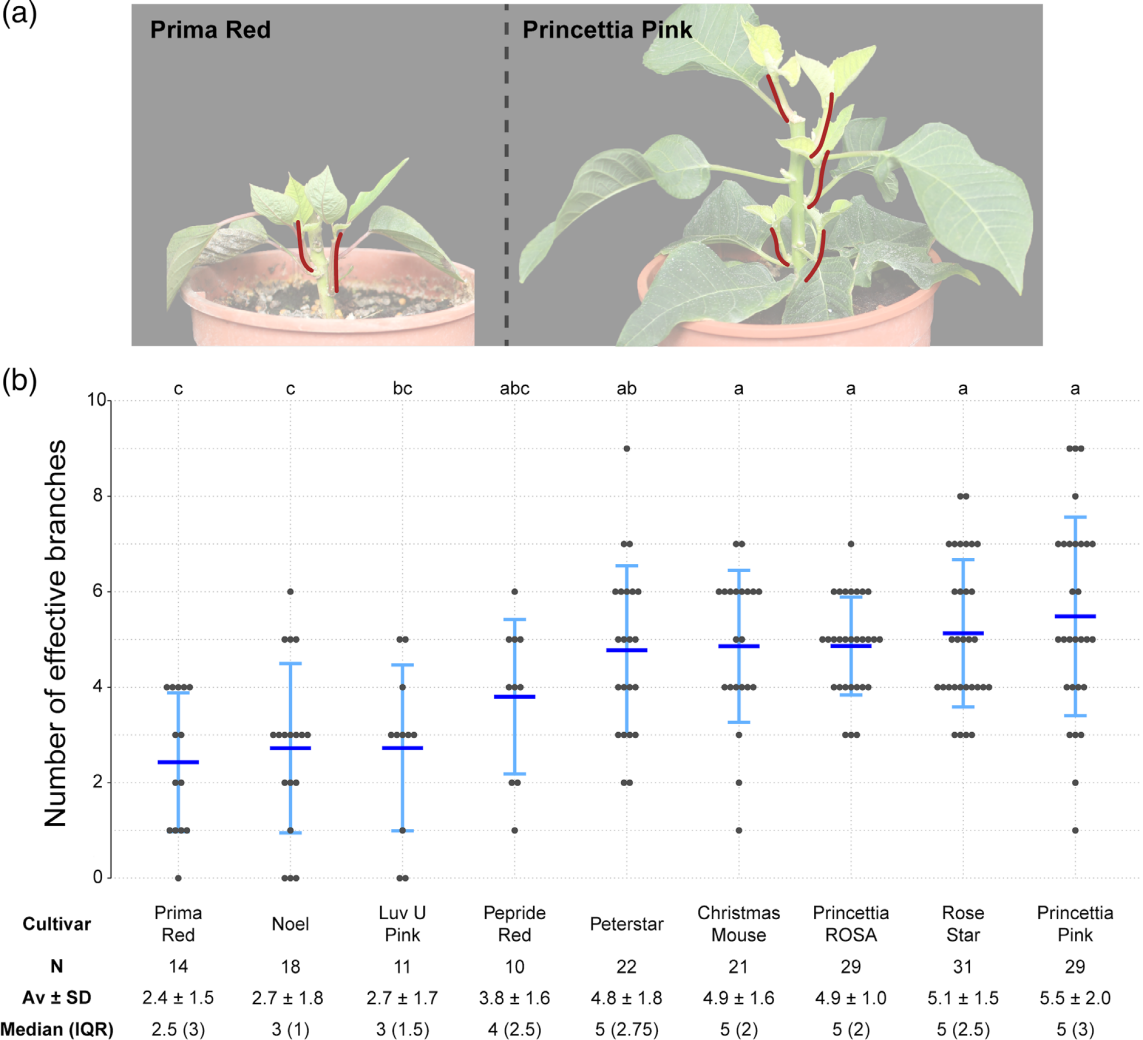
Branching performance among poinsettia cultivars. Poinsettia cuttings were pinched to exactly ten nodes, and branching performance was evaluated 3 weeks later. Newly formed branches ≥0.5 cm in length were counted as effective branches. (**a**) Representative images of the low-branching cultivar Prima Red and the high-branching cultivar Princettia Pink. Effective branches are highlighted in red. (**b**) Quantification of branching performance across nine cultivars. Each point represents an individual plant. *N* indicates the number of cuttings scored per cultivar. Average±sd are shown as horizontal bars in the plot and as numerical values below the x-axis. Median and interquartile range (IQR) are also provided. Statistical comparisons were performed using the Kruskal–Wallis test followed by Dunn’s post hoc tests. Different letters indicate significant differences at *P*<0.05 after multiple-testing correction. Adjusted *P*-values are provided in Table S4A, available in the online Supplementary Material 1.

To identify a PoiBI phytoplasma that is likely associated with a strong branching phenotype of its host, we compared the branching performance among nine poinsettia cultivars. These include Christmas Mouse, Luv U Pink, Noel, Pepride Red, Peterstar, Rose Star, Prima Red, Princettia Pink and Princettia ROSA, all from the collection maintained at the Taoyuan District Agricultural Research and Extension Station, Shulin Substation (New Taipei, Taiwan). For each cultivar, 32 cuttings containing 6 to 7 nodes were generated from maternal plants in May 2023. Wounding sites were treated with a phytohormone solution (1,500 mg l^−1^ indole-3-butyric acid and 500 mg l^−1^ 1-naphthaleneacetic acid) prior to planting in rockwool grow cubes to promote adventitious root formation. After new roots were formed, the plants were repotted into 5-inch pots and then maintained in a glass greenhouse at the substation. After the plants grew to have more than 10 nodes, they were pinched to leave exactly ten nodes. The branching performance was evaluated at 3 weeks after pinching. Plants were not randomized within the greenhouse, and branch counting was not performed blind. Newly formed axillary shoots ≥0.5 cm were counted to provide a predefined, length-based operational definition of effective branches. The number of plants successfully evaluated for each cultivar is indicated as *N* in Fig. 1.

### Phytoplasma genome analysis

The procedures of comparative genomics and phylogenetic analysis were largely based on those described in our previous studies [27,33, 34]. More detailed information is provided in the following sections. Unless stated otherwise, the methods were based on the cited references and the bioinformatic tools were used with the default settings.

For analysis of the PR2021 genome, the annotated assembly was obtained from the National Center for Biotechnology Information (NCBI) GenBank (accession GCA_029746895.1). All protein-coding genes were extracted from the GenBank record and assigned into COG functional categories [35] using BlastKOALA web tool [36] (Table S1, available in the online Supplementary Material 1). The chromosome map was drawn using Circos v0.69-6 [37]. To identify its close relatives, we obtained all 272 phytoplasma genome assemblies available from the NCBI as of 1 March 2025 (Table S2, available in the online Supplementary Material 1). Assemblies with N50 <10 kb were excluded to minimize the impact of highly fragmented genomes. Pairwise genome-wide average nucleotide identity (ANI) values were calculated using FastANI v1.33 [38]. Strains sharing ≥90% ANI with PR2021 were retained for comparative analyses.

After identification of strains that are closely related to PR2021, we referred to the ‘*Ca*. P.’ phylogenetic trees based on 16S rRNA gene [39] and core genome [27] to select other representative species for comparative analysis at the genus level. *Acholeplasma laidlawii* (GCA_000018785.1) was included as the outgroup. The genome sequences included in this analysis, together with the assembly statistics, are provided in Table S3 (available in the online Supplementary Material 1). The levels of completeness of these assemblies were estimated using BUSCO v6.0.0 with the ‘Phytoplasma’ and ‘Acholeplasmataceae’ datasets [40]. Homologous genes were identified using blastp v2.11.0 [41] with e-value cutoff set to 1e−15, followed by clustering of similarity results using OrthoMCL v1.3 [42]. Pseudogenes were omitted in the analysis.

To infer core genome phylogeny, homologous genes that are conserved as a single copy in all genomes analysed were used. For phylogenetic inference, multiple sequence alignments (MSAs) were prepared using muscle v3.8.31 [43] and visualized using JalView v2.11 [44]. Maximum likelihood phylogenies were inferred using PhyML v3.3 [45] and visualized using FigTree v1.4.4. phylip v3.697 [46] was used for bootstrap analysis. For comparison of effector gene content, we focused on phytoplasma effector families that have been experimentally validated to manipulate plant development in previous studies and examined homologous gene clustering results manually based on annotation.

For comparative analysis of potential mobile units (PMUs), which are important for the evolution of phytoplasma effectors [33,47, 48], methodology of PMU identification in PR2021 and selection of representatives for comparisons were based on that established previously [27]. Briefly, based on the knowledge that intact PMUs can be up to 30 kb in size, we first identified genomic regions with at least four PMU core genes (i.e. *tra5*, *dnaB*, *dnaG*, *tmk*, *hflB*, *himA*, *ssb* and *rpoD*) [49], each within ≤15 kb from the nearest neighbour. The PMU core genes located between two *tra5* homologues, which mark the PMU boundaries, were manually examined for their orientation to confirm that these genes belong to the same PMU. Visualization was performed using genoPlotR v0.8.11 [50].

### Bioinformatic analysis of SAP11 homologues

The methods for MSA and phylogenetic inference were based on those described for core genome phylogeny in the previous section. For a detailed examination of the protein sequences, the signal peptide was predicted using SignalP v5.0 [51]. Nuclear localization signal was predicted using two separate tools, with a positive result from either one considered as valid, including WoLF PSORT [52] webtool (organism type set to ‘Plant’) and PredictNLS v1.0.20 [53]. Coiled-coil domain was predicted using COILS-WRAP [54] with the settings ‘-m MTIDK -w 1’.

The method for protein structure prediction followed a recent study [55], which used AlphaFold2 [56] via ColabFold [57]. Prediction parameters included msa_mode, MMseqs2 (UniRef +Environmental); num_models, 5; num_recycles, 12; and stop_at_score, 100. For each protein, the model with the highest average predicted local distance difference test (pLDDT) score among the five generated was selected for visualization using web tool Mol* 3D Viewer [58]. To assess prediction confidence, we examined the MSA generated by ColabFold, including alignment depth and sequence identity to database entries. In addition, predicted aligned error (PAE) maps produced by AlphaFold2 were inspected to evaluate confidence in the relative positioning of residues within the predicted structures.

### Transient expression assays

For experimental investigation of putative effector genes, we performed transient expression assays in *N. benthamiana* [24,59]. The candidates include two SAP11 homologues identified in the genome of ‘*Ca*. P. pruni’ PR2021 (locus tags: PR2021_2540 and PR2021_3970). The codon-optimized sequences, designed for *N. benthamiana* and without the secretory signal peptide, were synthesized by GenScript (Piscataway, NJ, USA). The synthesized sequences were cloned into a potato virus X (PVX)-based expression vector pgR106 [60] using NEBuilder HiFi DNA Assembly (New England Biolabs). The resulting vectors were transformed into *Agrobacterium tumefaciens* strain GV3101 with pSOUP (Addgene 165419) via electroporation. The empty vector was included as the negative control.

For agroinfiltration in 3-week-old *N. benthamiana* plants, the agrobacterial strains harbouring the desired plasmids were cultured in lysogeny broth with rifampicin (50 µg ml^−1^), gentamicin (10 µg ml^−1^) and kanamycin (50 µg ml^−1^) for 48 h at 28 °C, harvested by centrifugation at 4,000 ***g*** for 15 min and resuspended in infiltration buffer (10 mM MgCl_2_, 10 mM MES, 100 µM acetosyringone and pH 5.7) with OD_600_ adjusted to 0.5. Three leaves of each plant were randomly selected to perform infiltration of 2 ml bacterial suspension. At 3 weeks after leaf infiltration, the number of side branches with at least three unfolded leaves for each plant was recorded. In each batch, five plants were used as biological replicates for each treatment. A total of three batches were performed during June–August 2025 using plant growth chambers in Academia Sinica (Taipei, Taiwan). Methods for statistical tests and visualization followed those described for poinsettia branching performance.

### Statistical tests and visualization of branching counts

The statistical significance was evaluated using the Kruskal–Wallis test followed by Dunn’s post hoc tests with Bonferroni correction for multiple comparisons, implemented with the packages stats v4.4.0 and dunn.test v1.3.6 in the R statistical environment v4.4.0 [61]. A non-parametric approach was chosen because pre-examination of branching count data with residual quantile–quantile plots and the Shapiro–Wilk test, implemented with stats v4.4.0, raised concern about violations of normality assumptions in both experiments. For the *N. benthamiana* experiment, the batch effect was tested using two-way ANOVA implemented with stats v4.4.0. Data visualization was performed using ggplot2 v3.5.1 [62], with all individual data points shown. The mean±sd was plotted for ease of interpretation, and letters assigned with multcompView v0.1–10 denote groups significantly different at *P*<0.05.

## Results and discussion

### Strong branching of Princettia Pink motivated genomic characterization of its phytoplasma

In our branching performance evaluation, the Kruskal–Wallis test followed by Dunn’s post hoc comparisons separated the nine cultivars into three overlapping significance groups (a–c) (Fig. 1, Table S4A available in the online Supplementary Material 1). The four top-performing cultivars were assigned to the highest group (a), while Prima Red and Noel were assigned to the lowest group (c). The remaining three cultivars fell into intermediate overlapping groups (ab, abc and bc), reflecting that they were not significantly different from at least one higher and one lower cultivar. The highest-performing cultivar, Princettia Pink, developed 5.5±2.0 effective branches at 3 weeks after pinching, which is notably higher than the ~2.4–2.7 range observed for the three lowest-performing cultivars.

This single-batch phenotyping experiment was intended to guide cultivar selection for genome sequencing rather than to establish a definitive ranking of branching performance. Phytoplasma titre, which is known to be positively correlated with branching [30], was not measured and therefore limits the interpretation of the observed differences. Despite the limitation, the performance difference was pronounced. Accordingly, we selected Princettia Pink as the target for genomic characterization of its associated phytoplasma, designated strain PR2021. The vigorous growth of this cultivar also provided sufficient leaf midribs, the preferred tissue for metagenomic sequencing targeting phloem-residing phytoplasmas.

As reported previously, we obtained the complete genome sequence of PoiBI phytoplasma strain PR2021 through a hybrid assembly approach [32]. The Illumina and ONT reads provided 107.8- and 115.5-fold sequencing depth, respectively. For the circularized chromosomal contig, BUSCO analysis recovered 200 of 202 phytoplasma conserved marker genes as single copy, corresponding to an estimated completeness of 99.0% (Table S3, available in the online Supplementary Material 1). To further investigate this finding, we conducted manual inspection based on tblastn sequence similarity searches against the PR2021 chromosomal contig. For translation initiation factor 3, a locus with 99% coverage and 99% amino acid sequence identity was identified; however, the nucleotides corresponding to the canonical start codon are altered to TAC in this strain, and this locus was therefore not predicted as a protein-coding sequence. For the GroEL-like apical domain superfamily protein, a locus with limited similarity (25% coverage and 43% amino acid sequence identity) was identified. It is therefore unclear whether this locus represents a highly divergent or pseudogenized homologue, or whether this conserved marker gene is absent in PR2021. Taken together, these observations are consistent with a high level of completeness of the PR2021 genome assembly.

### Comparative analysis of PR2021 and other phytoplasmas

**Fig. 2.**
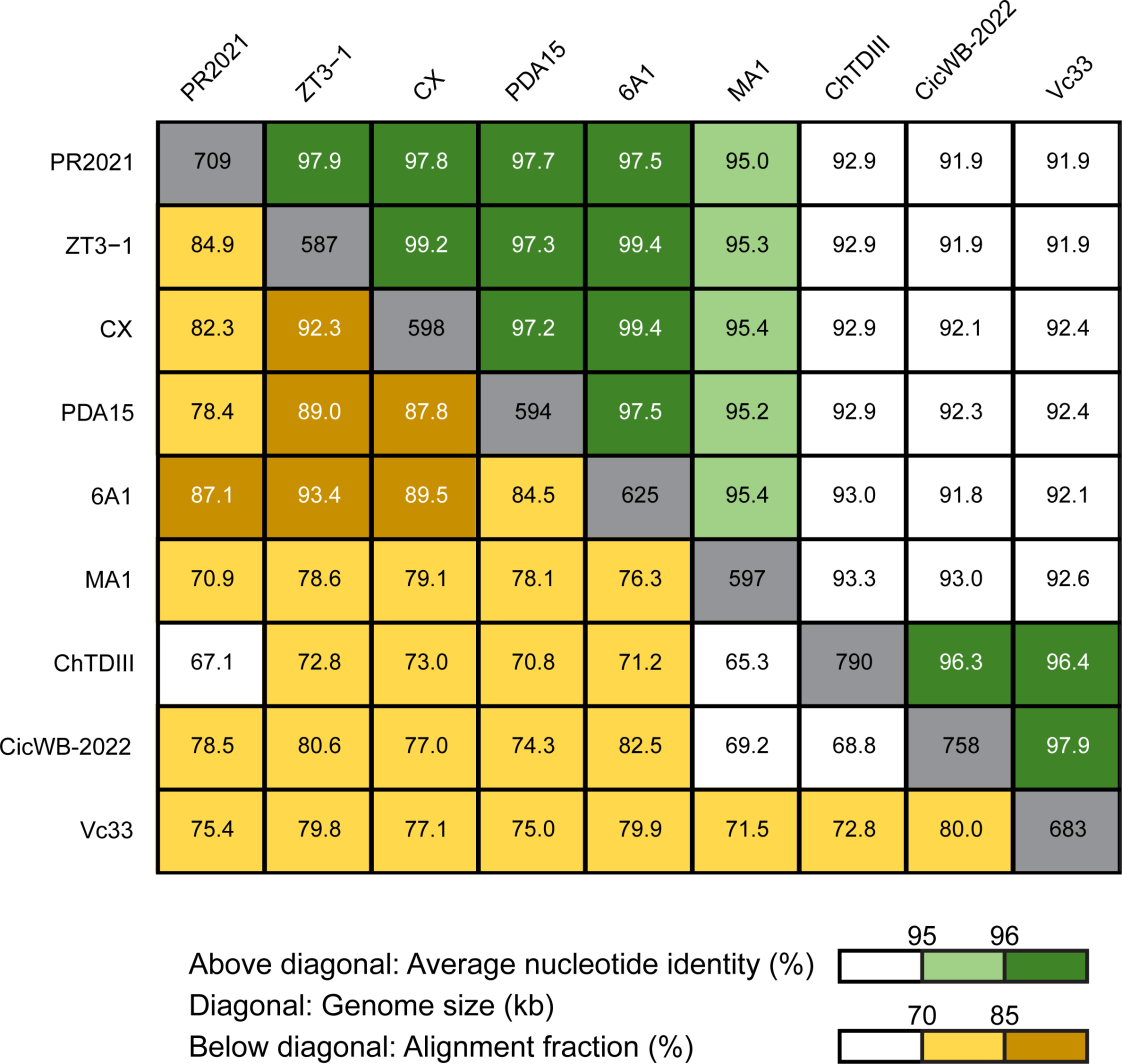
Pairwise genome similarity among ‘*Ca*. P. pruni’ PR2021 and closely related strains. The alignment fraction and ANI values for each pairwise comparison are provided below and above the diagonal, respectively. Values in the diagonal cells indicate genome sizes.

For comparative analysis of the PR2021 genome, we first identified its close relatives from publicly available assemblies (Table S2, available in the online Supplementary Material 1). Being uncultivated bacteria, phytoplasma genomes are typically reconstructed from metagenomic data. To avoid biases introduced by low-quality assemblies, we used assembly contiguity as a quality filter and excluded 49 highly fragmented assemblies with an N50 value below 10 kb. Considering the formal guideline of a 95% ANI threshold for delineating different ‘*Ca*. P.’ species [6], we applied a more inclusive cutoff of 90% ANI to capture closely related but potentially distinct taxa and identified eight strains (Fig. 2). Compared to these eight closely related strains, PR2021 shares 82.3% alignment fraction and 97.8% ANI with strain CX associated with Canada X-disease and described as ‘*Ca*. P. pruni’ [63,64], confirming its species assignment [6,32]. Meanwhile, although strain ChTDIII associated with chinaberry trees (*Melia azedarach* L.) in Argentina was initially identified as a member of ‘*Ca*. P. pruni’ [65], this strain, together with two other South American strains, CicWB-2022 from Argentina [66] and Vc33 from Chile [67], shares only ~92–93% ANI with strain CX and other members of ‘*Ca*. P. pruni’ (Fig. 2). Based on the 95% ANI threshold recommended in the latest guideline for delineating ‘*Ca*. P.’ species [6], these three South American strains are best regarded as representing a distinct species that requires further studies for a formal description.

**Fig. 3.**
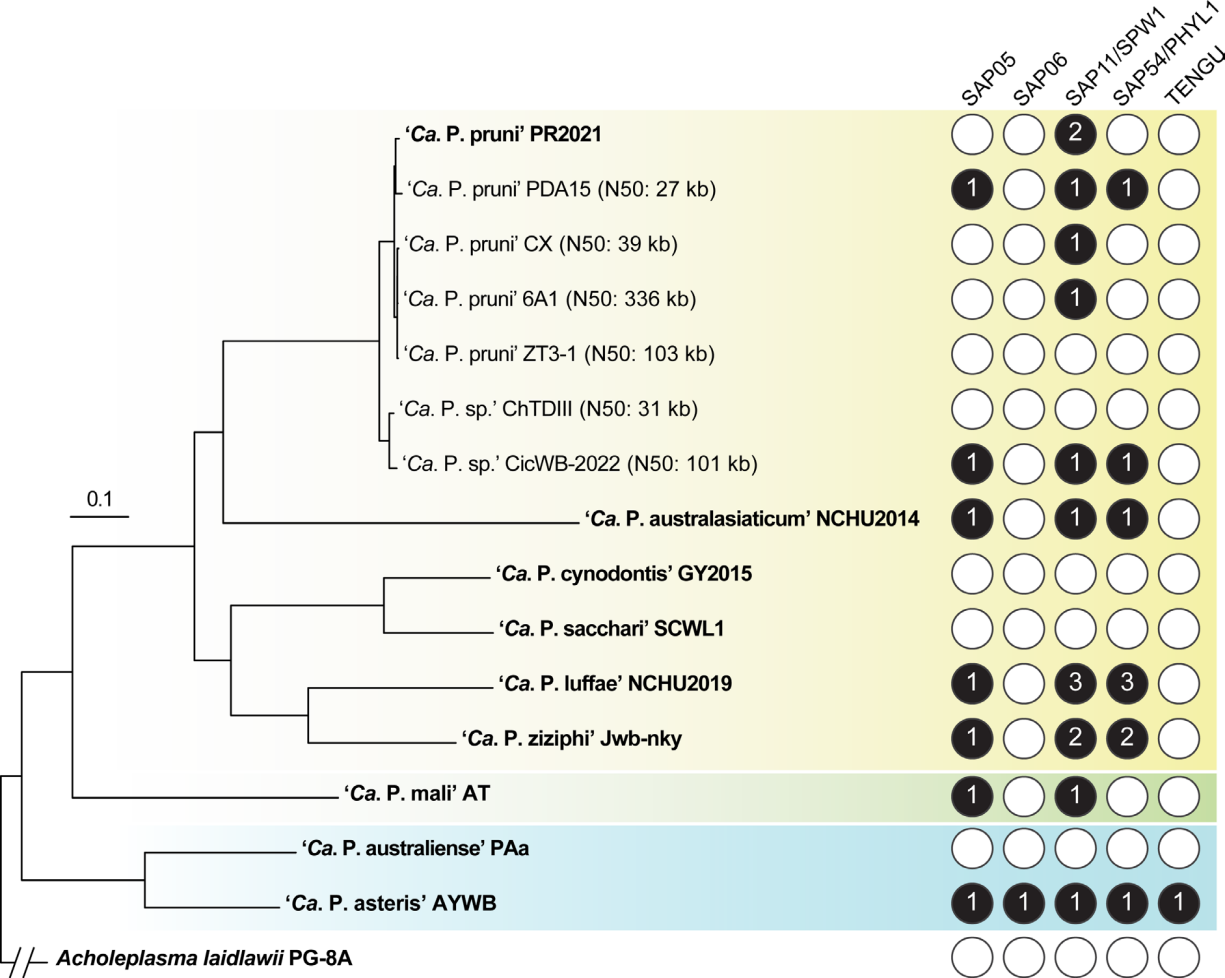
Core genome phylogeny and effector gene content. The maximum likelihood phylogeny was inferred using 163 conserved single-copy genes (Table S5, available in the online Supplementary Material 1), and the concatenated alignment contained 59,155 aligned amino acid sites. Based on 1,000 bootstrap resampling, the grouping of PR2021 and PDA15 received 61% support, all other internal nodes received >98% support. Strains with complete genome assemblies are highlighted in bold, and N50 values are provided in parentheses for strains with incomplete draft assemblies. The three major phylogenetic groups of phytoplasmas are indicated by a coloured background. *Acholeplasma laidlawii* was included as the outgroup. For the five characterized phytoplasma effectors, gene presence is indicated by filled circles, with copy number given inside; gene absence is indicated by empty circles.

For genus-level analysis, the phytoplasma genomes included for comparisons showed BUSCO completeness estimates ranging from 89.1 to 98.5%, with an average recovery of 95.6% of conserved marker genes (Table S3, available in the online Supplementary Material 1), consistent with generally high levels of assembly completeness. We inferred a core genome phylogeny based on 163 conserved single-copy genes with 59,155 aligned amino acid sites (Fig. 3, Table S5 available in the online Supplementary Material 1). Two PR2021-related strains, MA1 (197 contigs, N50=12 kb) and Vc33 (36 contigs, N50=36 kb) (Table S3, available in the online Supplementary Material 1), were excluded from the core genome analysis due to the lack of annotation in these assemblies. The phylogenetic placements further support the aforementioned species delineation, with strains with >95% ANI forming distinct monophyletic clades. Comparative analysis of effector gene content revealed that PR2021 has two SAP11 homologues, but no other previously characterized Sec-dependent phytoplasma effectors (Fig. 3). Given the complete circularized chromosomal contig of PR2021, these gene absences are unlikely to reflect false negative results caused by assembly artefacts. It is worth noting that a recent study examined non-classically secreted candidate effectors in ‘*Ca*. P. ziziphi’ and identified six proteins capable of suppressing plant hypersensitive response in heterologous assays [68]. While these findings highlight the potential breadth of phytoplasma–host interactions, the relevance of such candidates to specific developmental phenotypes, such as phytoplasma-induced branching, has yet to be established.

In PR2021, the presence of two SAP11 homologues is notable, because SAP11 homologues from diverse phytoplasmas have been reported to induce branching phenotypes. The effector SAP11 was first reported from ‘*Ca*. P. asteris’ strain AYWB and known to target plant nuclei [14]. Transgenic expression of this gene in *Arabidopsis thaliana* increases stem numbers, reportedly through the destabilization of plant TEOSINTE-BRANCHED, CYCLOIDEA, PROLIFERATION FACTOR 1 and 2 (TCP) transcription factors [10,11]. Follow-up studies for transgenic expression of different phytoplasma SAP11 homologues in *A. thaliana* [13,16] and *N. benthamiana* [15] reported similar phenotypes and mechanisms. Based on these previous studies, the SAP11 homologues found in PR2021 are likely linked to the branching phenotype of its poinsettia host, therefore warranting further investigation.

### SAP11 homologues in PR2021 differ in genomic context and evolutionary origin

**Fig. 4.**
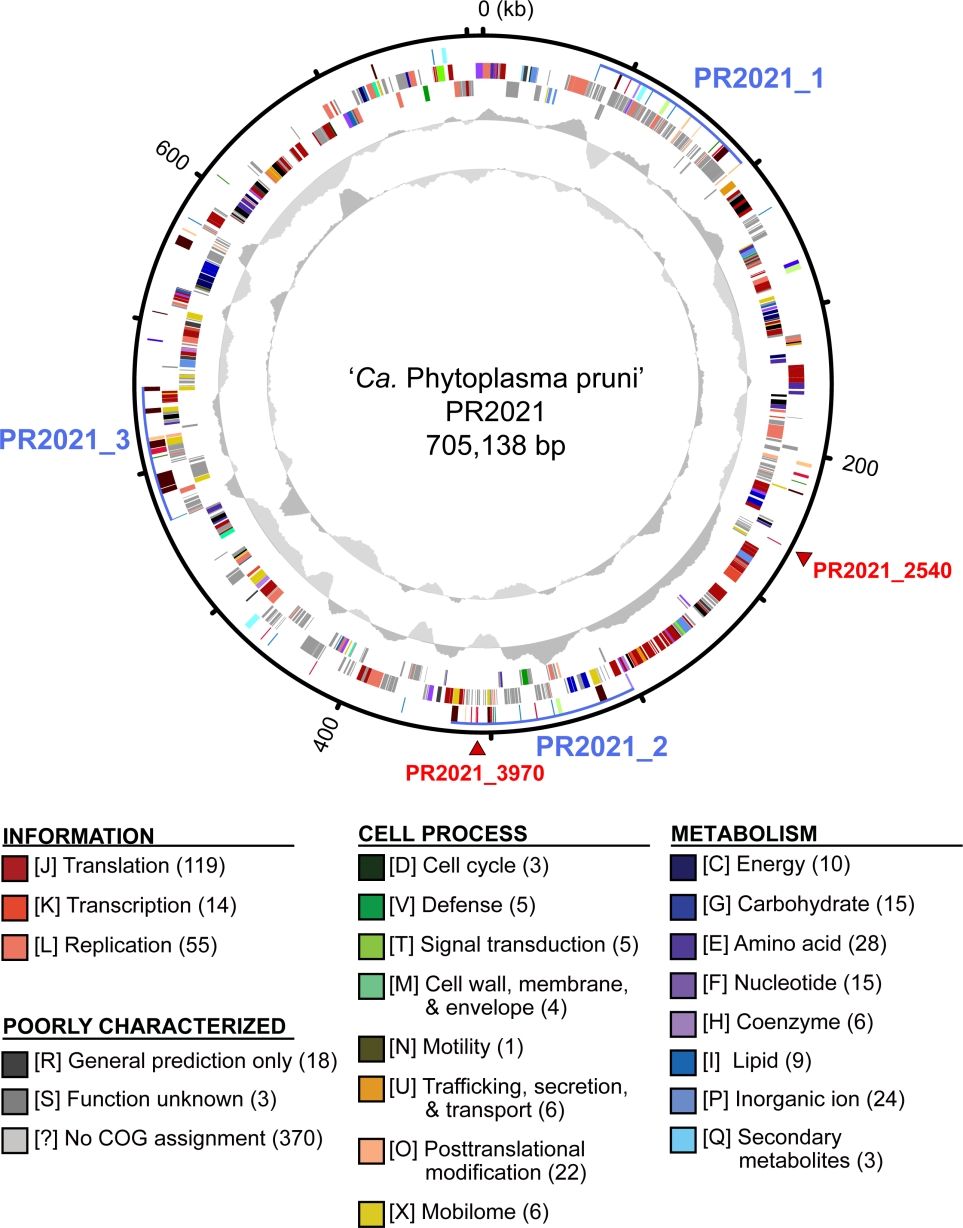
Chromosome map of ‘*Candidatus* Phytoplasma pruni’ PR2021. Concentric rings from outside in the following: (1) scale marks (kb). (2) Genes associated with PMUs or annotated as encoding putative secreted proteins; colour-coded according to the scheme illustrated in Fig. 5. The three gene clusters corresponding to intact PMUs are highlighted by blue lines. PMU identifiers and the two SAP11 homologues are indicated with blue and red labels outside of the scale mark ring, respectively. (3 and 4) Coding sequences on the forward and reverse strand, respectively. Colour-coded by functional categories; gene counts for each category are shown in the legend, with detailed information provided in Table S1 (available in the online Supplementary Material 1). (5) GC skew (positive, dark grey; negative, light grey). (6) GC content (above average, dark grey; below average, light grey).

**Fig. 5.**
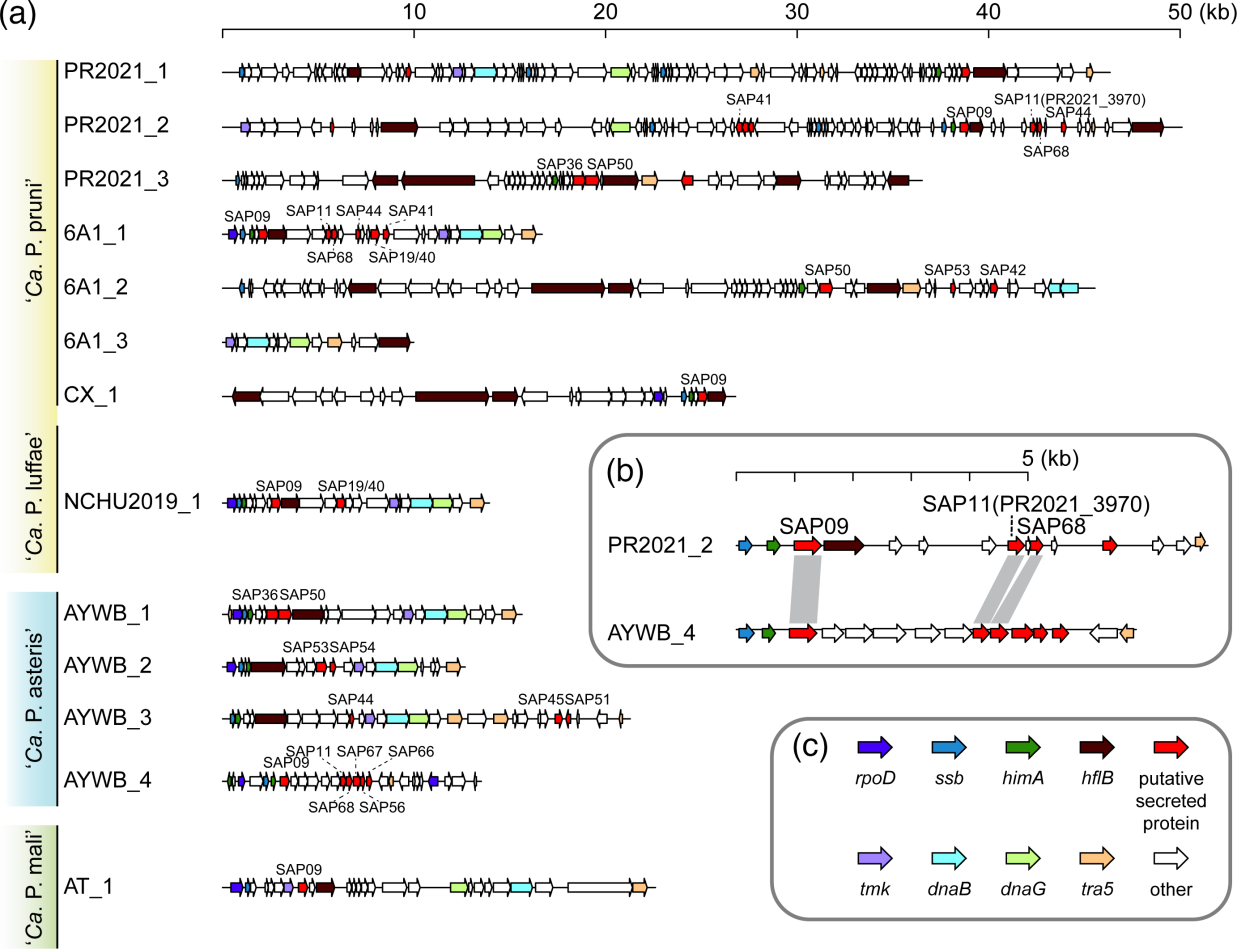
Gene organization of representative PMUs. (**a**) Representative PMUs selected from diverse phytoplasmas. Defined PMUs are labelled with the phytoplasma strain name and a numerical identifier. Coding sequences in the defined regions were drawn to scale, with their orientations indicated by arrows. Homologues of previously described secreted AYWB proteins (SAP) are labelled. (**b**) An enlarged view comparing the regions containing SAP11 homologues between PR2021_2 and AYWB_4. (**c**) Colour codes for PMU core genes and genes encoding putative secreted proteins.

Intriguingly, the two SAP11 homologues in PR2021 have distinct genomic contexts. One homologue (locus tag: PR2021_2540; GenBank protein ID: WEK82326.1) is 17.7 kb away from the nearest PMU gene and 69.0 kb away from the nearest intact PMU; therefore, it is not considered as PMU-associated (Fig. 4). By contrast, the other SAP11 homologue (PR2021_3970; WEK82465.1) is clearly located within a long PMU, designated as PR2021_2 (Figs 4 and 5), suggesting distinct evolutionary histories for these two homologues.

Comparisons of PMUs among representative phytoplasmas revealed that ‘*Ca*. P. pruni’ harbours usually long PMUs. PR2021, which has a complete genome assembly, contains three intact PMUs ranging from 45 to 62 kb. Strain 6A1, which has a high-quality assembly with an N50 value of 336 kb, harbours three PMUs ranging from 12 to 56 kb. For strain CX, although only one 33 kb PMU was identified, PMU content could not be accurately assessed for this highly fragmented assembly with an N50 value of 39 kb. Together, compared to the size range of 6–28 kb observed in previously defined PMUs across diverse phytoplasmas [27] (Fig. 5), the newly characterized ‘*Ca*. P. pruni’ genomes revealed that phytoplasma PMU diversity is higher than previously recognized.

Our closer inspection of the PMU PR2021_2 revealed that PR2021_3970 is co-localized with several other SAP homologues, including SAP09 and SAP68 (Fig. 5b). A similar association among these genes was found in the PMU AYWB_4 from ‘*Ca*. P. asteris’, which contains the first experimentally characterized SAP11 [10,14]. Although PR2021_2 and AYWB_4 share little overall synteny conservation, possibly due to the long evolutionary distance between ‘*Ca*. P. pruni’ and ‘*Ca*. P. asteris’ (Fig. 3), the similarity of this SAP gene set supports a role for PMUs in horizontal gene transfer across divergent phytoplasma lineages. The involvement of PMUs in the evolution of phytoplasma genomes and effector genes has been implicated across multiple species and effector families [27,33, 48]. However, it is also worth noting that several well-characterized effectors, including SAP05 [8], SAP06 [9] and TENGU [24] in ‘*Ca*. P. asteris’, are located outside PMU regions, indicating that PMU association is not a universal feature of phytoplasma effectors.

**Fig. 6.**
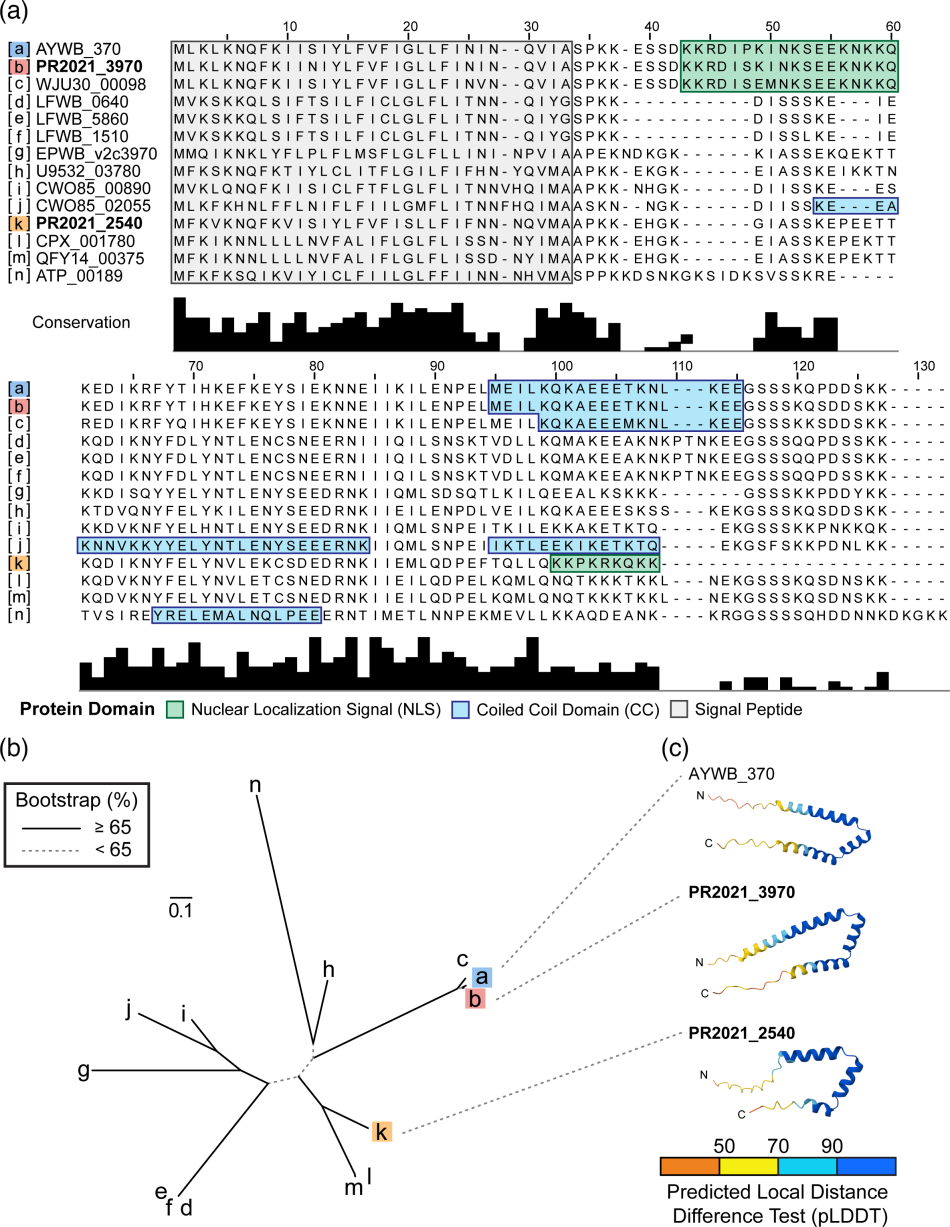
Bioinformatic analysis of SAP11 homologues. (**a**) MSA. All homologues are identified by the locus tags from their respective genome annotation. The two homologues found in the PR2021 genome are highlighted in bold. Predicted domains are highlighted with coloured backgrounds. (**b**) Maximum likelihood phylogeny. Bootstrap support levels were assessed based on 1,000 resampling. Homologues are labelled with lowercase alphabets as illustrated in panel (a). (**c**) Protein structure prediction of the three key homologues, including the previously studied SAP11 from ‘*Ca*. P. asteris’ AYWB (locus tag: AYWB_370) and the two homologues identified in the PR2021 genome. Regions of the predicted structures are colour-coded according to the confidence values based on pLDDT.

Sequence- and structure-based comparisons further highlight the contrasting evolutionary histories of the two PR2021 SAP11 homologues. The protein sequence encoded by PR2021_3970 shares 97.5% amino acid identity to ‘*Ca*. P. asteris’ SAP11 (locus tag: AYWB_370) (Fig. 6a) and clusters closely with it in phylogenetic analyses (Fig. 6b), despite the long divergence between the two species (Fig. 3). AlphaFold2 structural predictions also yielded nearly indistinguishable models (Fig. 6c). These observations are consistent with PR2021_3970 having been acquired recently through horizontal transfer, likely mediated by PMUs. By contrast, PR2021_2540 is highly divergent from AYWB_370, sharing only 39.0% identity and 47.9% similarity in their protein sequences (Fig. 6a). It shares 56.0–56.9% identity and 68.1% similarity to two other SAP11 homologues found in other ‘*Ca*. P. pruni’ strains, namely, CPX_001780 from strain CX and QFY14_00375 from strain 6A1. These findings, together with the non-PMU chromosomal location of PR2021_2540, suggested that PR2021_2540 represents a vertically inherited homologue retained within ‘*Ca*. P. pruni’. This inference raises the question of whether these two evolutionarily distinct homologues are functionally equivalent.

### Both SAP11 homologues are sufficient to induce branching in *N. benthamiana*

The finding that PR2021_3970 is nearly identical to the experimentally characterized AYWB_370, both in their amino acid sequences and predicted protein structures (Fig. 6), makes this SAP11 homologue a strong candidate for explaining the branch-inducing property of PoiBI phytoplasmas. However, whether the second SAP11 homologue PR2021_2540 also plays a functional role is unclear. On the one hand, it exhibits substantial sequence divergence and shows lower similarity in predicted protein structure. On the other hand, its predicted structure also contains three alpha helices, as observed in PR2021_3970 and AYWB_370, with consistently high local confidence (pLDDT >90) (Fig. 6c). Although the multiple sequence alignments for these proteins were shallow with ~30 hits (Fig. S1A, available in the online Supplementary Material 2), as expected given their genus-restricted distribution, the central helical regions of all three proteins exhibited low PAE (<5 Å), consistent with stable relative positioning of residues within this region (Fig. S1B, available in the online Supplementary Material 2). Together, these results support confidence in the predicted three-alpha-helix fold at the local level in the central region of all three homologues. The conservation of this central three-alpha-helix fold in PR2021_2540, despite substantial sequence divergence, raised the possibility that it may retain SAP11-associated activity, motivating functional testing *in planta*.

**Fig. 7.**
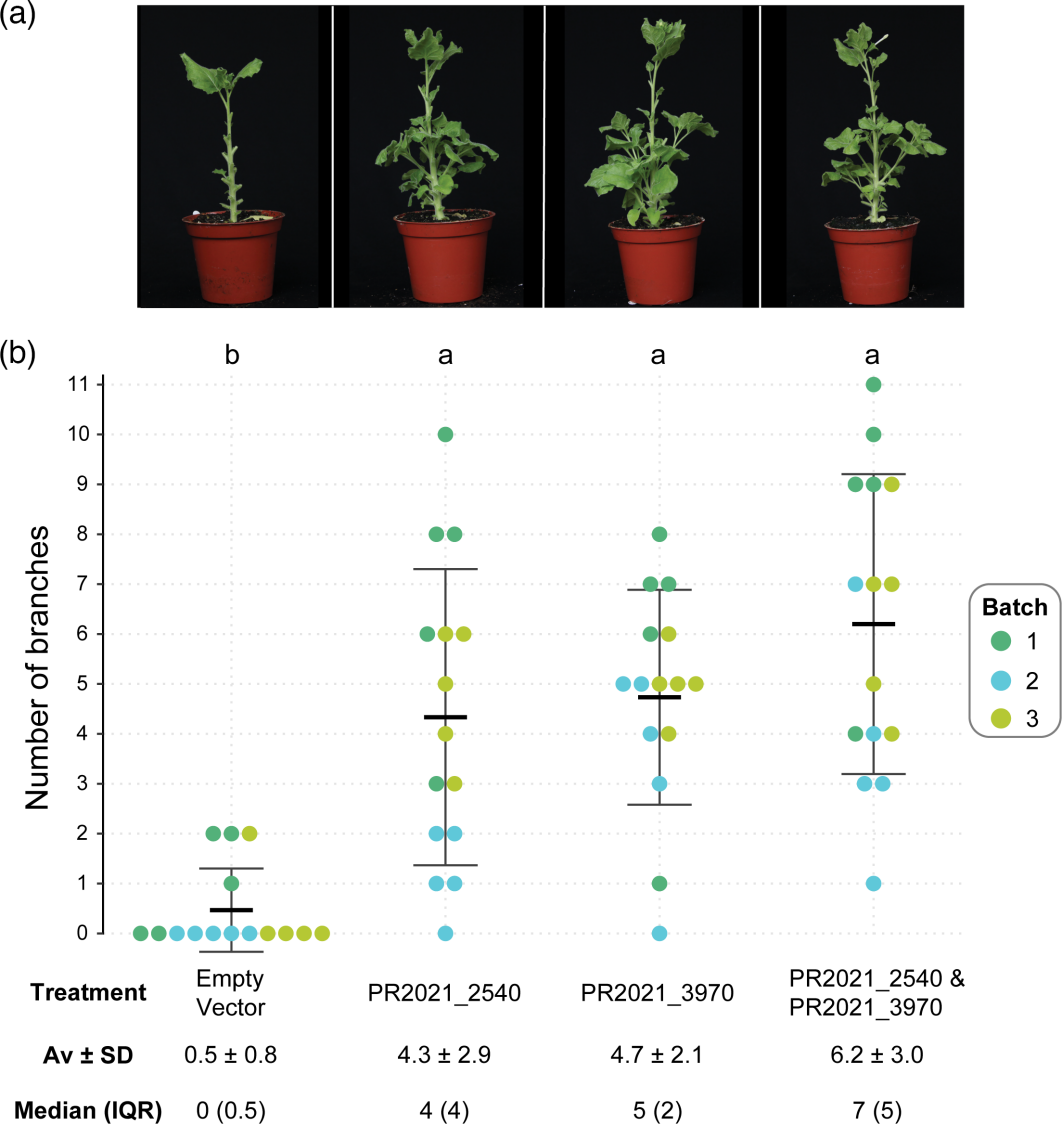
Experimental characterization of PR2021-encoded SAP11 homologues. SAP11 homologues were expressed in 3-week-old *N. benthamiana* by agroinfiltration. Side branches with at least three unfolded leaves were counted 3 weeks post-infiltration. Fully unfolded leaves were removed prior to imaging to facilitate branch counting. Each treatment included 15 plants in total, with 5 biological replicates per batch across 3 independent batches. (**a**) Photos of representative plants from each treatment. (**b**) Quantification of branch counts. Each point represents an individual plant. Average±sd are shown as horizontal bars in the plot and as numerical values below the x-axis. Median and interquartile range (IQR) are also provided. Statistical comparisons were performed using the Kruskal–Wallis test followed by Dunn’s post hoc tests. Different letters indicate significant differences at *P*<0.05 after multiple-testing correction. Adjusted *P*-values are provided in Table S4B (available in the online Supplementary Material 1).

To empirically test if these two SAP11 homologues found in PR2021 are capable of inducing branching in host plants, we used a PVX-based transient expression system to express codon-optimized versions of these two genes, both individually and jointly, in *N. benthamiana*. At 3 weeks post-agroinfiltration, control plants transformed with the empty vector were mostly unbranched, whereas the expression of either homologue alone induced ~4–5 side branches per plant, and co-expression averaged ~6 branches (Fig. 7). Among the three independent batches, plants from batch 1 had more branches than those from batch 3 across all treatments, but two-way ANOVA did not detect a statistically significant batch effect (*P*=0.061).

Although the expression levels of these genes were not quantified, qualitatively similar branching phenotypes were observed across three independent batches. Rank-based comparisons showed that all three treatments differed significantly from the negative control, while not differing from one another (Table S4B, available in the online Supplementary Material 1). Thus, in this transient expression assay, each SAP11 homologue is independently sufficient to induce branching. The activity of PR2021_3970 is consistent with its near identity to the AYWB SAP11 (Fig. 6) previously characterized in *Arabidopsis* [10,11], whereas the comparable activity of the highly divergent, non-PMU PR2021_2540 is notable. The lack of significant additivity in co-expression is consistent with action on overlapping host targets or saturation of the branching response. Mechanistic links to TCP destabilization have been established for other SAP11 homologues [10,11, 13, 15,17] but were not tested here.

Together with the genomics and sequence analyses above, these results show that PR2021 harbours two distinct SAP11 homologues, both of which are functional in the transient expression assay used here. The coexistence of two homologues may provide functional redundancy, which could buffer against gene loss or permit differential regulation under specific conditions. However, the available data do not allow us to determine whether retention of both homologues confers a measurable fitness advantage.

## Conclusion

In this study, we characterized the genome of the poinsettia-associated phytoplasma PR2021 and linked its effector repertoire to the free-branching trait of its host. Comparative genomics confirmed PR2021 as ‘*Ca*. P. pruni’ and revealed unusually long PMUs distinct from previously defined types. This genome encodes two SAP11 homologues, one likely retained through vertical inheritance and the other acquired through horizontal transfer, but no other known phytoplasma effectors. Functional assays demonstrated that each homologue is independently sufficient to induce branching in *N. benthamiana*. Notably, the branch-inducing property of PR2021_2540, despite its high sequence divergence from other SAP11 homologues, reveals functional conservation within this effector family that was not previously recognized. The absence of additive effects between the two homologues suggests functional redundancy, which may contribute to robust host manipulation. Beyond explaining an unusual plant–pathogen interaction with horticultural benefit, our results, together with prior work, highlight TCP transcription factors as promising targets for breeding or synthetic effector-based approaches to engineer branching traits without reliance on phytoplasma infection.

## Supplementary material

10.1099/mgen.0.001675 Supplementary Material 1.

10.1099/mgen.0.001675 Supplementary Material 2.

## Abbreviations

ANI: average nucleotide identity
MSA: multiple sequence alignment
ONT: Oxford Nanopore Technologies
PAE: predicted aligned error
pLDDT: predicted local distance difference test
PMU: potential mobile unit
PoiBI: poinsettia branch-inducing
PVX: potato virus X
