# Supplementary material for: Comparative genomics of a poinsettia-associated phytoplasma and functional validation of its SAP11-homologous effectors that induce plant branching
Source: Microb Genom. 2026 Mar 30;12(3):001675. doi: 10.1099/mgen.0.001675 (PMC13293309; doi:10.1099/mgen.0.001675)
Supplement: Supplementary Material 2. [file mgen-12-01675-s002.pdf]

SUPPLEMENTARY MATERIALS

Comparative genomics of a poinsettia-associated phytoplasma and functional validation of its SAP11-homologous effectors that induce plant branching

Shen-Chian Pei[1,2], Nian-Pu Li[1,2], Ting-Ting Li[3], Ya-Ching Yang[3], Ting-Hsuan Hung[2]*, Chih-Horng Kuo[1]*

[1] Institute of Plant and Microbial Biology, Academia Sinica, Taipei 115201, Taiwan

[2] Department of Plant Pathology and Microbiology, National Taiwan University, Taipei 106319, Taiwan

[3] Taoyuan District Agricultural Research and Extension Station - Shulin Substation, Ministry of Agriculture, New Taipei 238014, Taiwan

* Correspondence:

Ting-Hsuan Hung; thhung@ntu.edu.tw

Chih-Horng Kuo; chk@gate.sinica.edu.tw

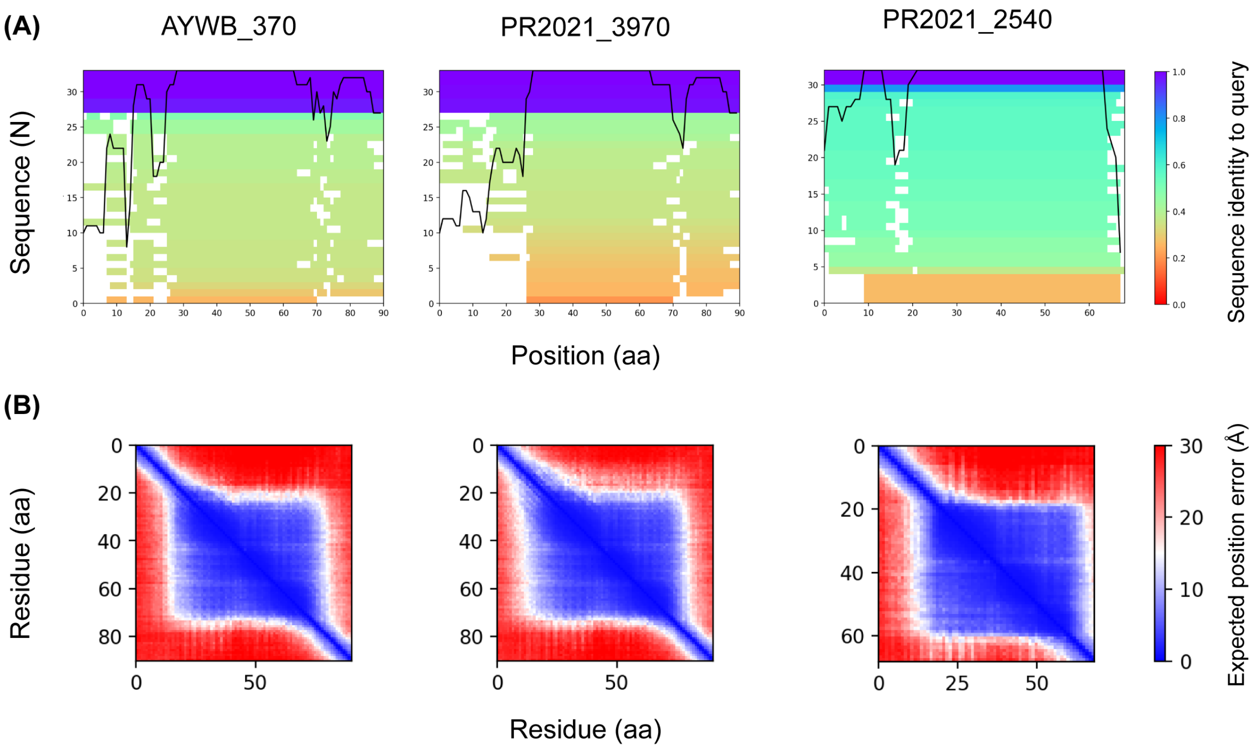

Fig. S1. Confidence assessment of AlphaFold2 structure predictions for SAP11 homologs. (A) Multiple sequence alignment (MSA) depth and sequence identity distributions for each protein, as generated by ColabFold using the MMseqs2 (UniRef + Environmental) database. The limited MSA depths reflect the genus-restricted distribution of SAP11 homologs in current sequence databases. (B) Predicted aligned error (PAE) maps for the selected AlphaFold2 models. Low PAE values (< 5 Å) are observed in the central regions corresponding to the predicted three–alpha-helix fold, indicating stable relative positioning of residues within this region.

Additional Supplementary Materials (available as a separate .xlsx file):

Table S1. List of protein-coding genes in PR2021 genome.

Table S2. List of the phytoplasma genome assemblies available from NCBI as of March 1, 2025.

Table S3. List of the genome assemblies included in comparative analysis.

Table S4. Adjusted p-values from multiple pairwise comparisons. (A) Branching performance among poinsettia cultivars (Fig. 1). (B) Branching effects induced by putative effectors (Fig. 7).

Table S5. List of core genes used for phylogeny. (A) '*Ca*. P. pruni' PR2021 (GCA_029746895.1). (B) '*Ca*. P. pruni' PDA15 (GCA_041984945.1). (C) '*Ca*. P. pruni' CX (GCA_001277135.1). (D) '*Ca*. P. pruni' 6A1 (GCA_033391615.1). (E) '*Ca*. P. pruni' ZT3-1 (GCA_024586395.1). (F) '*Ca*. P. sp.' ChTDIII (GCA_013391955.1). (G) '*Ca*. P. sp.' CicWB-2022 (GCA_035853675.1). (H) '*Ca*. P. australasiaticum' NCHU2014 (GCA_001307505.2). (I) '*Ca*. P. cynodontis' GY2015 (GCA_030127385.1). (J) '*Ca*. P. sacchari' SCWL1 (GCA_027594465.1). (K) '*Ca*. P. luffae' NCHU2019 (GCA_018024475.1). (L) '*Ca*. P. ziziphi' Jwb-nky (GCA_003640545.1). (M) '*Ca*. P. mali' AT (GCA_000026205.1). (N) '*Ca*. P. australiense' PAa (GCA_000069925.1). (O) '*Ca*. P. asteris' AYWB (GCA_000012225.1). (P) *Acholeplasma laidlawii* PG-8A (GCA_000018785.1).
